# Factors Associated With Behavioral and Psychological Symptoms of Dementia: Prospective Observational Study Using Actigraphy

**DOI:** 10.2196/29001

**Published:** 2021-10-29

**Authors:** Eunhee Cho, Sujin Kim, Sinwoo Hwang, Eunji Kwon, Seok-Jae Heo, Jun Hong Lee, Byoung Seok Ye, Bada Kang

**Affiliations:** 1 Mo-Im Kim Nursing Research Institute College of Nursing Yonsei University Seoul Republic of Korea; 2 College of Nursing Yonsei University Seoul Republic of Korea; 3 Korea Armed Forces Nursing Academy Daejeon Republic of Korea; 4 Department of Biostatistics and Computing Yonsei University Graduate School Yonsei University Seoul Republic of Korea; 5 National Health Insurance Service Ilsan Hospital Goyang Republic of Korea; 6 College of Medicine Yonsei University Seoul Republic of Korea

**Keywords:** behavioral and psychological symptoms, dementia, older adults, actigraphy, sleep, activity, risk factors

## Abstract

**Background:**

Although disclosing the predictors of different behavioral and psychological symptoms of dementia (BPSD) is the first step in developing person-centered interventions, current understanding is limited, as it considers BPSD as a homogenous construct. This fails to account for their heterogeneity and hinders development of interventions that address the underlying causes of the target BPSD subsyndromes. Moreover, understanding the influence of proximal factors—circadian rhythm–related factors (ie, sleep and activity levels) and physical and psychosocial unmet needs states—on BPSD subsyndromes is limited, due to the challenges of obtaining objective and/or continuous time-varying measures.

**Objective:**

The aim of this study was to explore factors associated with BPSD subsyndromes among community-dwelling older adults with dementia, considering sets of background and proximal factors (ie, actigraphy-measured sleep and physical activity levels and diary-based caregiver-perceived symptom triggers), guided by the need-driven dementia-compromised behavior model.

**Methods:**

A prospective observational study design was employed. Study participants included 145 older adults with dementia living at home. The mean age at baseline was 81.2 (SD 6.01) years and the sample consisted of 86 (59.3%) women. BPSD were measured with a BPSD diary kept by caregivers and were categorized into seven subsyndromes. Independent variables consisted of background characteristics and proximal factors (ie, sleep and physical activity levels measured using actigraphy and caregiver-reported contributing factors assessed using a BPSD diary). Generalized linear mixed models (GLMMs) were used to examine the factors that predicted the occurrence of BPSD subsyndromes. We compared the models based on the Akaike information criterion, the Bayesian information criterion, and likelihood ratio testing.

**Results:**

Compared to the GLMMs with only background factors, the addition of actigraphy and diary-based data improved model fit for every BPSD subsyndrome. The number of hours of nighttime sleep was a predictor of the next day’s sleep and nighttime behaviors (odds ratio [OR] 0.9, 95% CI 0.8-1.0; *P*=.005), and the amount of energy expenditure was a predictor for euphoria or elation (OR 0.02, 95% CI 0.0-0.5; *P*=.02). All subsyndromes, except for euphoria or elation, were significantly associated with hunger or thirst and urination or bowel movements, and all BPSD subsyndromes showed an association with environmental change. Age, marital status, premorbid personality, and taking sedatives were predictors of specific BPSD subsyndromes.

**Conclusions:**

BPSD are clinically heterogeneous, and their occurrence can be predicted by different contributing factors. Our results for various BPSD suggest a critical window for timely intervention and care planning. Findings from this study will help devise symptom-targeted and individualized interventions to prevent and manage BPSD and facilitate personalized dementia care.

## Introduction

Behavioral and psychological symptoms of dementia (BPSD) constitute a core and prevalent feature of Alzheimer disease and related dementia [1], with most patients experiencing one or more types of symptoms over the course of the disease [2]. BPSD refer to frequently occurring symptoms of disturbed perception through content, mood, or behavior [3], which manifest into a wide range of forms, such as agitation, aggression, depression, apathy, wandering, and socially inappropriate behaviors [1]. Increasingly recognized as the most challenging aspect of dementia, BPSD present adverse outcomes, including decreased functioning and accelerated disease progression [4], and, if poorly managed, an increased risk of nursing home placement [5] and hospitalization [4,6,7]. BPSD are also associated with an increased burden on, and decreased quality of life of, caregivers [8-10].

Although BPSD are associated with neurological mechanisms of neurocognitive disease to some extent, previous studies have revealed that the actual occurrence of symptoms can be attributed to diverse personal factors (eg, medical conditions, premorbid personality, and physical and psychological unmet needs), social factors (eg, communication with caregivers, caregivers’ stress and depression, and lack of social activities), and environmental factors (eg, overstimulation and lack of established routines), rather than to neurocognitive impairment alone [11,12]. While a few studies have provided evidence that supports frequent co-occurrence of individual BPSD, others have contended that BPSD are distinct and heterogeneous and have different determinants and consequences [13-15]. Determining key factors that predict the different types of BPSD is the first step, since it would guide the determination of which strategies should be chosen to target the underlying causes and ultimately prevent or manage the symptoms [12,16,17].

We based this study on the need-driven dementia-compromised behavior (NDB) model [18]. In the NDB model, BPSD arise from the interaction of two types of factors: (1) relatively stable background factors, including sociodemographic characteristics; neurological, cognitive, and functional status; underlying health; and personality traits, and (2) proximal factors, which are fluctuating and changing states of physical and psychological unmet needs and immediate environmental conditions. To further explain stress-related proximal factors, we incorporated the progressively lowered stress threshold (PLST) model [19], which posits that individuals with dementia are increasingly unable to manage stress, as the threshold for stress tolerance lowers as the disease progresses. If heightened perceived stressors accumulate, then exceed the stress threshold, the person with dementia starts to exhibit BPSD [19-21]. The PLST model recognizes circadian rhythms as a factor that influences the stress threshold level and accordingly postulates that impaired sleep and inadequate physical activity level are among the stressors that consequently trigger BPSD [22,23].

Previous studies have recommended person-centered nonpharmacological interventions as the first-line treatment modality for managing BPSD given the limited efficacy and undesired adverse effects of antipsychotics [24-26]. However, the effect size of the existing nonpharmacological interventions for BPSD has been small [27]. Thus, caregivers and providers continue to struggle to implement the most effective interventions targeting the underlying cause of certain types of BPSD due to limited evidence for target symptoms [28]. Moreover, current knowledge of BPSD is limited, since most existing studies considered BPSD imprecisely as a unitary construct by using measures that aggregated symptoms, which failed to account for the heterogeneity of different types of BPSD [12,14,29]. Although a range of factors have been associated with BPSD occurrence in the literature, most studies focused on only one aspect by testing the effect of a single intervention, such as music therapy, environmental modification, or structured recreational activities [30,31]. A recent scoping review highlighted a gap in the research, namely that diverse factors, including personal, social, and environmental, have been studied only for depression [12]. Surprisingly, few predictive models in existing studies accounted conjointly for the diverse factors [32,33].

A few studies found that sleep problems are associated with depression [34,35], apathy [35,36], agitation [37,38], and aggression [39]; however, efforts have been limited by the use of proxy-rated instruments for disturbed sleep [34,35,39]. Since caregiver-reported sleep measures require systemic and continuous observation of sleep behaviors, such a resource-intensive and time-consuming assessment may be infeasible in real-time care settings [40]. Further, as Blytt et al found significant discrepancies between proxy-rated sleep and actigraphy measures, proxy raters may underreport and fail to recognize sleep disturbances as compared with those measured by actigraphy [41].

Although physical activity has increasingly gained attention as a nonpharmacological approach to managing BPSD [42], few studies have examined the influence of physical activity on BPSD using observational rating instruments to measure activity level [43,44]. A study reported that physical activity objectively measured using actigraphy was significantly correlated with agitation and aggression but did not account for other potential factors [45]. In summary, surprisingly little is known about the influence of circadian rhythms, including sleep and physical activity levels, on BPSD among community-dwelling older adults with dementia. This is due to the limited number of methodologically rigorous studies using objective measures, such as actigraphy, and accounting for personal, social, and environmental factors within a single study.

Understanding factors that predict BPSD subsyndromes is important not only for establishing early care planning for symptom prevention and management but for selecting the most effective person-centered interventions. Thus, this study explored factors associated with BPSD subsyndromes among community-dwelling older adults with dementia, considering background and proximal factors (ie, actigraphy-measured sleep and physical activity levels and diary-based caregiver-perceived symptom triggers), guided by the NDB model.

## Methods

### Design

This exploratory study employed a prospective observational design with two waves of data collection. Within each wave, background factors were collected at baseline, and repeated measures were collected for proximal factors over approximately 14 days. Reporting of this study adheres to the Strengthening the Reporting of Observational Studies in Epidemiology (STROBE) statement [46].

### Participants and Setting

Older adults with dementia living at home were recruited from outpatient neurology clinics at two tertiary hospitals and daycare centers in Seoul and the Gyeonggi region in Korea. Inclusion criteria for older adults with dementia were as follows: aged at least 65 years, a diagnosis of dementia by a physician, a Mini–Mental State Examination (MMSE) score of less than 24, and exhibition of BPSD at least once a week, as screened using the Korean version of the Neuropsychiatric Inventory at baseline [47]. The primary caregivers who provided the majority of care for the recruited older adults with dementia, lived in the same home, and were able to read and write in Korean were also included.

### Procedure

The Institutional Review Board of the affiliated institutions approved this study. Participants were recruited via on-site visits between June 2018 and June 2019. We conducted the second-wave data collection for participants who agreed to continue in the study between July 2019 and June 2020. The assessment and data collection were conducted by research staff that included registered nurses with a master’s degree and registered nurse research assistants with a bachelor’s degree. The research staff with a master’s degree taught the data collection protocol and trained the research assistants in on-site data collection. The research staff then contacted potential participants and explained the study purpose and procedures. All data collection was conducted at the participants’ homes. After obtaining written informed consent at baseline from caregivers who were screened for eligibility, family caregivers completed a structured questionnaire consisting of sociodemographic information and standardized scales for physical and neuropsychological assessments, with assistance from the research staff. Following the baseline assessments, we placed an actigraphy device on the participants’ wrists, and primary caregivers logged the BPSD manifestations into the BPSD diary on a daily basis for 14 consecutive days.

### Measures

#### Outcome Measure: BPSD

Although the Neuropsychiatric Inventory [48] is a widely used tool to measure BPSD, its 2-week retrospective rating likely results in recall bias, since its rating reliability is dependent on caregiver training [49]. Therefore, we developed a BPSD diary, adapted from the Neuropsychiatric Inventory, to detect 12 behavioral and psychological symptoms commonly observed in patients with dementia on a daily basis: delusions, hallucinations, agitation or aggression, depression or dysphoria, anxiety, elation or euphoria, apathy or indifference, disinhibition, irritability or lability, aberrant motor behavior, sleep and nighttime behavior, and appetite or eating disorders. The BPSD diary is a structured, easy-to-use checklist that allows a caregiver to record the presence and severity (ie, mild, moderate, and severe) of individual symptoms.

Although BPSD encompass heterogeneous symptoms, clustering a number of individual symptoms that are highly correlated and contingently co-occur allows for a more meaningful interpretation of the study findings [17,50]. Moreover, use of subsyndromes rather than 12 individual symptoms can increase power, as the number of participants who endorse the symptom cluster will increase [51]. Since euphoria or elation, aberrant motor behavior, sleep and nighttime behavior, and appetite or eating disorders did not load on any clusters in previous studies [50-54], we included them as individual symptoms. The remaining eight symptoms were clustered as follows, based on results from factor analyses reported in the literature: (1) psychotic symptoms (hallucination and delusion), (2) affective symptoms (depression, anxiety, and apathy), and (3) hyperactivity (agitation or aggression, disinhibition, and irritability) [51,53-55].

#### Proximal Factors

##### Sleep and Physical Activity

Sleep and physical activity were objectively measured using actigraphy (the wGT3X-BT activity monitor; ActiGraph, LLC). Wrist actigraphy has been shown to be a reliable method by which to objectively measure sleep-wake cycles and is suitable for older adults with dementia [56,57]. Participants were asked to wear actigraphy devices on their nondominant wrist for 14 consecutive days starting on the day of the first visit. ActiLife software (version 6.13.3; ActiGraph, LLC) was used to evaluate actigraphy data and provide standard indices of sleep duration and fragmentation using vector magnitude counts in 60-second epoch data. We applied the Cole-Kripke algorithm to score a 1-minute epoch as asleep or awake [58]. We defined nighttime sleep as the time between 8 PM and 8 AM. We used total sleep time (TST) at night and wake time after sleep onset (WASO) at night as sleep parameters. The previous night’s sleep parameters were used as potential predictors of BPSD occurring the following day. We also used actigraphy-derived energy expenditure (kcal burned × 100/hour) as a parameter of activity level. Energy expenditure measured for 24 hours was used as a potential predictor of BPSD occurring on the same day, which reflected the physical conditions during the day when BPSD were exhibited.

##### Physiological Unmet Needs States and Interpersonal and Environmental Triggers

We used a checklist, embedded within the BPSD diary, to assess contributing factors that family caregivers considered to be immediate triggers for symptoms that encompassed physiological unmet needs states and interpersonal and environmental triggers. On the checklist, the triggers were specifically listed as follows: hunger or thirst, urination or bowel movement, pain or discomfort, sleep disturbance, noise, light, temperature, interpersonal trigger related to a person or persons who were present when the symptom occurred, environmental change, and other. Caregivers were asked to check all contributing factors that were present on the same day when the BPSD were present on this daily-basis symptom checklist. When caregivers checked the “other” option, they were asked to provide a brief description of the triggers. Diary-based caregiver-reported contributing factors that were observed immediately prior to BPSD occurrence were used as potential predictors of BPSD occurring on the same day.

#### Background Factors

##### Sociodemographic and Health Information

Participants provided demographic data, including age, gender, marital status, and education level. Dementia diagnosis and neurological and psychiatric medications were obtained via medical chart review or interviews with family caregivers and staff at the recruitment sites.

##### Cognitive and Functional Status

The Korean version of the MMSE (K-MMSE) was administered to evaluate cognitive functioning at baseline. The highest total score is 30 points, with a higher score indicating better cognitive function [59]. The Cronbach α score for Korean older adults with dementia was .91 [60]. The Korean version of the Activities of Daily Living scale (K-ADL) was administered to measure baseline functional status. This measure consists of seven items that are rated on a 3-point Likert scale, with higher scores indicating more severe dependency. The K-ADL has been validated for Korean older adults with dementia, exhibiting good psychometric properties (eg, Cronbach α score of .94) [61].

##### Personality Type

A family caregiver informant was asked to rate his or her family member’s premorbid personality using the Korean version of the Big Five Inventory (BFI-K) [62]. The BFI-K consists of 15 items that are rated on a 5-point Likert scale, which assess five domains of personality features, namely openness, conscientiousness, neuroticism, extroversion, and agreeableness. The instrument is known to be reliable, with Cronbach α scores ranging from .67 to .82 in a Korean sample [62].

##### Statistical Analysis

For descriptive statistics, categorical variables were expressed as the number of dementia patients by percentage, while continuous variables were presented as means and SDs. We used two-sample independent *t* tests and Fisher exact tests to compare the difference between data from waves 1 and 2 (Tables S1-S3 in Multimedia Appendix 1). We used generalized linear mixed models (GLMMs) to explore factors predictive of BPSD subsyndromes, with estimation of odds ratios (ORs) and 95% CIs. The GLMM, a commonly used random-effects model, was well suited to this analysis because the approach permits random effects and is suitable for nonnormal or discrete outcomes that are repeatedly measured for each subject [63,64]. All models included a random effect of participant with a random intercept to account for heterogeneity among individuals; all other factors were modeled as fixed effects [65]. Once we decided on the variables to be included in the final model based on clinical and theoretical relevance, we then calculated variance inflation factors to assess multicollinearity among variables. We found that the data were suitable for regression analyses, given that all variance inflation factors were <5, which indicated that no multicollinearity could be detected (Multimedia Appendix 2). The GLMMs to predict each BPSD subsyndrome (ie, psychotic symptoms, affective symptoms, hyperactivity, euphoria or elation, aberrant motor behavior, sleep and nighttime behaviors, and appetite or eating disorders) were tested and compared. Predictors were included in the models in a block-wise manner: (1) background factors (age, gender, marital status, education, K-ADL score, K-MMSE score, premorbid personality type, taking sedative, and dementia type) and (2) proximal factors (TST at night, WASO at night, and caregiver-reported symptom triggers). The order in which the blocks entered the model was determined based on clinical and theoretical considerations: background factors entered first (Model 1), followed by proximal factors (Model 2) for each BPSD subsyndrome. Given that all available data from each participant in the data sets from waves 1 and 2 were used to fit the GLMM, a time variable that indicated the wave (wave 1 vs wave 2) was also present in the models. Fit of the models was assessed and compared using the Akaike information criterion (AIC), the Bayes information criterion (BIC), and the likelihood ratio test. Results were statistically significant if *P*<.05. All analyses were performed using R (version 3.6.3; The R Foundation).

## Results

### Background Characteristics

During the first wave of data collection, we initially recruited 166 eligible participants. Out of these participants, 18 (10.8%) were lost to follow-up due to refusal to wear the actigraphy devices (n=10), hospitalization or emergency room visits (n=6), death (n=1), and absence of family caregivers at home during the study period (n=1). A total of 3 (1.8%) participants were excluded from the analysis due to no available actigraphy data. During the second wave of data collection, out of 64 eligible participants, 5 (8%) were excluded from the analysis due to no available actigraphy data (n=3) and no available BPSD diary data (n=2). Consequently, this study included a total of 145 older adults with dementia who participated in the first wave. Of the 145 participants, 59 (40.7%) older adults with dementia continued to participate in the second wave.

At baseline for the first wave, the participants’ mean age was 81.23 (SD 6.01) years, with a female to male ratio of approximately 3:2 (86/145, 59.3% female). Education level for most participants was elementary school or below. Participants had moderate cognitive impairment, as indicated by the K-MMSE mean score of 17.28 (SD 5.5); the mean score of the K-ADL was 10.57 (SD 3.6). Background characteristics of the 145 participants in the first wave at baseline are illustrated in Table 1; these characteristics are presented separately for the first and second waves in Table S1 in Multimedia Appendix 1.

**Table 1:** Background characteristics of older adults with dementia in the first wave at baseline.

Variable		Value (N=145)
Age (years), mean (SD)		81.23 (6.01)
**Gender, n (%)**		
	Male	59 (40.7)
	Female	86 (59.3)
**Marital status, n (%)**		
	Married	86 (59.3)
	Bereaved or divorced	59 (40.7)
**Education level, n (%)**		
	Elementary school or below	73 (50.3)
	Middle school	14 (9.7)
	High school	34 (23.4)
	College or above	24 (16.6)
Total K-ADL^a^ score, mean (SD)		10.57 (3.63)
Total K-MMSE^b^ score, mean (SD)		17.28 (5.51)
**Big Five Inventory score, mean (SD)**		
	Openness	8.60 (2.96)
	Conscientiousness	11.66 (2.73)
	Neuroticism	7.74 (2.87)
	Extroversion	8.49 (1.87)
	Agreeableness	10.92 (2.96)
Sedative (yes), n (%)		51 (35.2)
**Dementia type, n (%)**		
	Alzheimer disease	71 (49.0)
	Lewy body dementia	60 (41.4)
	Vascular dementia	23 (15.9)
	Other dementia	31 (21.4)

^a^K-ADL: Korean version of the Activities of Daily Living scale.

^b^K-MMSE: Korean version of the Mini–Mental State Examination.

### Descriptive Statistics for BPSD Occurrence and Proximal Factors

Summary statistics of BPSD and proximal factors for the 2354 days that encompassed the first and second waves are presented in Table 2; these factors are presented separately for the first and second waves in Table S2 in Multimedia Appendix 1. Caregivers completed the BPSD diaries and participants wore actigraphy devices for a mean of 11.5 (SD 3.5) days. Of the 2354 days during which the symptoms were measured, the most frequently occurring BPSD subsyndrome was affective symptoms (n=548, 23.3%), followed by hyperactivity symptoms (n=350, 14.9%) and sleep and nighttime behaviors (n=275, 11.7%). Euphoria or elation (n=108, 4.6%) and aberrant motor behaviors (n=103, 4.4%) were relatively infrequent. The prevalence of BPSD across participants is also presented in Table S3 in Multimedia Appendix 1. The prevalence of affective symptoms was the highest (82/145, 56.6%), followed by hyperactivity (70/145, 48.3%).

The mean TST at night was 6.4 (SD 2.5) hours, and the mean WASO at night was 0.4 (SD 0.4) hours. The mean energy expenditure (100 kcal/hour) was 0.21 (SD 0.14). Among the diverse set of caregiver-reported contributing factors, the most frequently reported factor was sleep disturbance (276/2354, 11.7%). Urination or bowel movement (220/2354, 9.3%), pain or discomfort (190/2354, 8.1%), and interpersonal triggers (171/2354, 7.3%) were also relatively frequent.

**Table 2:** Summary statistics of BPSD subsyndromes and proximal factors.

BPSD^a^ subsyndromes and proximal factors					Value (N=2354 days)
Days of recording BPSD and actigraphy per person, mean (SD)					11.54 (3.50)
**BPSD subsyndromes, n (%)**					
	Psychotic symptoms			234 (9.9)	
	Affective symptoms			548 (23.3)	
	Hyperactivity symptoms			350 (14.9)	
	Euphoria or elation			108 (4.6)	
	Aberrant motor behavior			103 (4.4)	
	Sleep and nighttime behavior			275 (11.7)	
	Appetite or eating disorders			193 (8.2)	
**Proximal factors**					
	**Sleep- and energy-related factors, mean (SD)**				
		Total sleep time (hours at night)	6.44 (2.52)		
		Wake time after sleep onset (hours at night)	0.43 (0.36)		
		Energy expenditure (100 kcal/hour)	0.21 (0.14)		
	**Caregiver-reported contributing factors, n (%)**				
		Hunger or thirst	149 (6.3)		
		Urination or bowel movement	220 (9.3)		
		Pain or discomfort	190 (8.1)		
		Sleep disturbance	276 (11.7)		
		Noise	76 (3.2)		
		Light	69 (2.9)		
		Temperature	103 (4.4)		
		Interpersonal trigger	171 (7.3)		
		Environmental change	87 (3.7)		
		Other	238 (10.1)		

^a^BPSD: behavioral and psychological symptoms of dementia.

### GLMMs Predicting BPSD Subsyndromes From Background and Proximal Factors

In the first step, the GLMMs for each BPSD subsyndrome were assessed with only background factors included in the models (Model 1; see results in Tables S1 and S2 in Multimedia Appendix 3). Based on Model 1, proximal factors—actigraphy-measured sleep and activity levels and diary-based caregiver-reported symptom triggers—were entered into Model 2. Table 3 presents *P* values of the likelihood ratio tests as well as the respective AIC and BIC scores for Models 1 and 2 for each BPSD subsyndrome. Likelihood ratio testing comparing Model 1, which consisted of only background factors, with Model 2 (ie, the full model), which included both background and proximal factors, showed that the full model had superior fit for every subsyndrome (*P*<.001). Goodness-of-fit statistics (ie, lower AIC and BIC values) indicated that models for every subsyndrome improved with the addition of proximal factors.

**Table 3:** Comparisons of generalized linear mixed models.

Outcomes (BPSD^a^ subsyndromes)	Background factors (Model 1)		Background factors + proximal factors (Model 2)		*P* value^b^
	AIC^c^	BIC^d^	AIC	BIC	
Psychotic symptoms	872.94	993.98	756.84	952.81	<.001
Affective symptoms	1683.85	1804.89	1379.99	1575.96	<.001
Hyperactivity symptoms	1278.41	1399.46	1064.87	1260.84	<.001
Euphoria or elation	689.12	810.17	587.68	783.65	<.001
Aberrant motor behaviors	525.33	646.37	442.19	638.16	<.001
Sleep and nighttime behaviors	1215.43	1336.47	957.22	1153.20	<.001
Appetite or eating disorders	882.84	1003.88	792.87	988.84	<.001

^a^BPSD: behavioral and psychological symptoms of dementia.

^b^*P* values were calculated by the likelihood ratio test.

^c^AIC: Akaike information criterion.

^d^BIC: Bayesian information criterion.

### Factors Predictive of BPSD Subsyndromes

**Figure 1:**
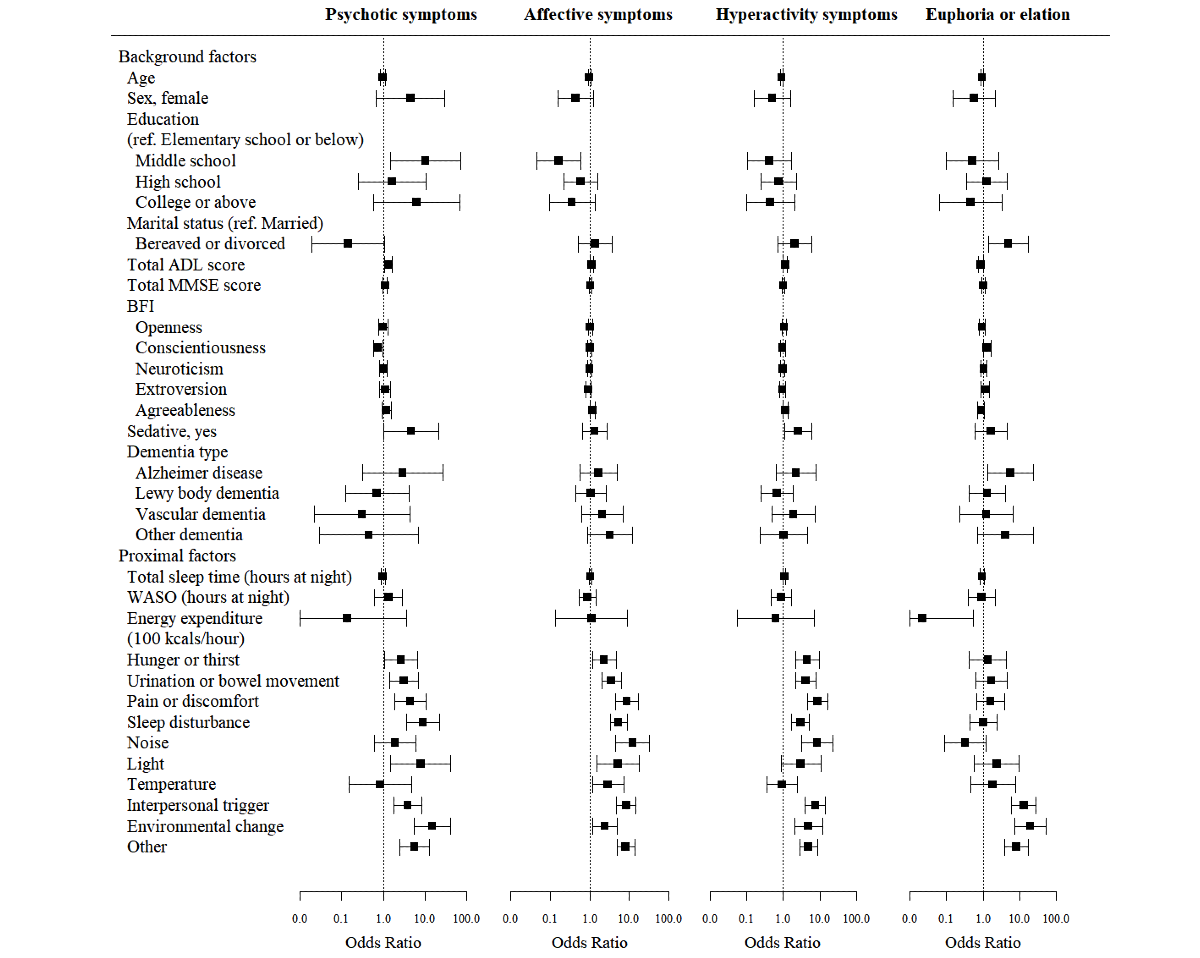
Forest plots of odds ratios (with 95% CIs shown as whiskers) for the influence of background and proximal factors on psychotic symptoms, affective symptoms, hyperactivity symptoms, and euphoria or elation. ADL: Activities of Daily Living scale; MMSE: Mini–Mental State Examination; BFI: Big Five Inventory; ref.: reference; WASO: wake time after sleep onset.

**Figure 2:**
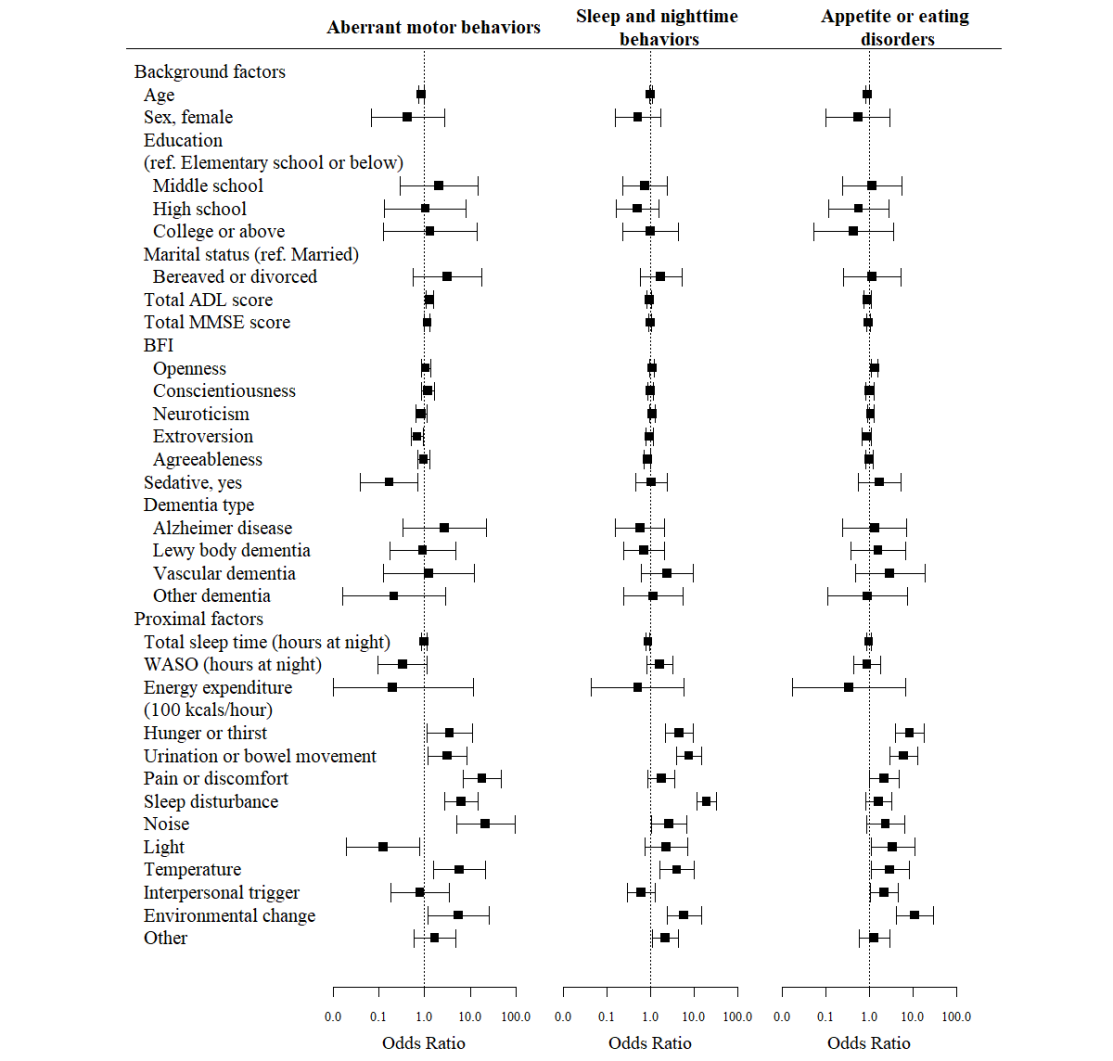
Forest plots of odds ratios (with 95% CIs shown as whiskers) for the influence of background and proximal factors on aberrant motor behavior, sleep and nighttime behaviors, and appetite or eating disorders. ADL: Activities of Daily Living scale; MMSE: Mini–Mental State Examination; BFI: Big Five Inventory; ref.: reference; WASO: wake time after sleep onset.

The results of the GLMMs of background and proximal factors as predictors of BPSD subsyndromes are depicted in forest plots of ORs in Figures 1 and 2 and displayed in Tables S1 and S2 in Multimedia Appendix 4. The results are also summarized below for each subsyndrome.

#### Psychotic Symptoms

Regarding proximal factors, patients were more likely to exhibit psychotic symptoms on the same day if they were exposed to environmental change (OR 14.7, 95% CI 5.3-40.8; *P*<.001) or inadequate light (OR 7.8, 95% CI 1.5-40.7; *P*=.02). Patients were more likely to exhibit psychotic symptoms (OR 8.8, 95% CI 3.6-21.7; *P*<.001) if they had sleep disturbance observed and reported by caregivers as well as pain or discomfort (OR 4.4, 95% CI 1.9-10.3; *P*<.001), urination or bowel movement–related problems (OR 3.1, 95% CI 1.4-6.9; *P*=.005), or hunger or thirst (OR 2.6, 95% CI 1.1-6.4; *P*=.03).

Background factors significantly associated with psychotic symptoms were educational attainment at the middle school level (OR 10.2, 95% CI 1.5-70.1; *P*=.02), greater impairment in activities of daily living (ADLs) (OR 1.3, 95% CI 1.1-1.7; *P*=.01), taking sedatives (OR 4.6, 95% CI 1.0-21.1; *P*=.049), and high conscientiousness traits (OR 0.8, 95% CI 0.6-1.0; *P*=.02).

#### Affective Symptoms

Of environmental condition–related proximal factors, increased likelihood of affective symptoms was significantly associated with noise (OR 12.1, 95% CI 4.5-32.4; *P*<.001), inadequate light (OR 5.1, 95% CI 1.5-17.4; *P*=.01), inadequate temperature (OR 2.9, 95% CI 1.2-7.1; *P*=.02), and being exposed to environmental change (OR 2.4, 95% CI 1.2-4.9; *P*=.02). Of proximal factors associated with physical unmet needs states, affective symptoms were more likely to be exhibited by patients with pain or discomfort (OR 8.6, 95% CI 4.6-16.4; *P*<.001), sleep disturbance (OR 5.3, 95% CI 3.3-8.7; *P*<.001), urination or bowel movement problems (OR 3.5, 95% CI 2.0-6.1; *P*<.001), or hunger or thirst (OR 2.3, 95% CI 1.2-4.6; *P*=.02). Interpersonal triggers related to a person or persons who were present with the patients with dementia were also significantly associated with an increased likelihood of affective symptoms (OR 8.4, 95% CI 4.8-14.6; *P*<.001).

Background factors significantly associated with affective symptoms included agreeable personality traits (OR 1.2, 95% CI 1.0-1.3; *P*=.03), greater impairment in ADLs (OR 1.1, 95% CI 1.0-1.3; *P*=.03), and educational attainment at the middle school level (OR 0.2, 95% CI 0.0-0.6; *P*=.005).

#### Hyperactivity Symptoms

With respect to proximal factors, patients were more likely to exhibit hyperactivity symptoms if they experienced pain or discomfort (OR 8.8, 95% CI 4.6-16.9; *P*<.001), hunger or thirst (OR 4.5, 95% CI 2.1-9.5; *P*<.001), urination or bowel movement problems (OR 4.2, 95% CI 2.2-8.0; *P*<.001), or sleep disturbance (OR 3.0, 95% CI 1.7-5.2; *P*<.001). Increased likelihood of hyperactivity symptoms was also predicted by being exposed to noise (OR 8.5, 95% CI 3.2-22.6; *P*<.001) and environmental change (OR 4.9, 95% CI 2.1-11.8; *P*<.001). Interpersonal triggers also increased the predicted odds of hyperactivity symptoms (OR 7.6, 95% CI 4.0-14.4; *P*<.001).

Background factors that increased the predicted odds of hyperactivity symptoms were greater impairment in ADLs (OR 1.1, 95% CI 1.0-1.3; *P*=.04) and taking sedatives (OR 2.5, 95% CI 1.1-5.9; *P*=.03).

#### Euphoria or Elation

Regarding proximal factors, patients with greater levels of energy expenditure (100 kcal/hour) measured by actigraphy were less likely to exhibit euphoria or elation on the same day (OR 0.02, 95% CI 0.0-0.5; *P*=.02). In contrast, patients who were exposed to environmental change (OR 19.7, 95% CI 7.5-51.7; *P*<.001) or interpersonal triggers (OR 13.0, 95% CI 6.0-28.4; *P*<.001) were more likely to show symptoms of euphoria or elation.

Background factors significantly associated with euphoria or elation included diagnosis of Alzheimer disease (OR 5.7, 95% CI 1.4-23.6; *P*=.02), bereavement or divorced status (OR 4.9, 95% CI 1.4-17.1; *P*=.01), and high conscientiousness traits (OR 1.3, 95% CI 1.0-1.6; *P*=.046).

#### Aberrant Motor Behaviors

Of proximal factors, environmental condition–related factors that increased the likelihood of aberrant motor behaviors were being exposed to noise (OR 21.6, 95% CI 5.0-93.5; *P*<.001), inadequate temperature (OR 5.8, 95% CI 1.6-21.3; *P*=.008), and environmental change (OR 5.6, 95% CI 1.2-26.0; *P*=.03). In contrast, light was significantly associated with decreased likelihood of such behaviors (OR 0.1, 95% CI 0.0-0.8; *P*=.03). Patients were more likely to exhibit BPSD behaviors if they had pain or discomfort (OR 18.0, 95% CI 6.9-47.1; *P*<.001), sleep disturbance (OR 6.4, 95% CI 2.7-14.9; *P*<.001), hunger or thirst (OR 3.6, 95% CI 1.1-11.2; *P*=.03), or urination or bowel movement problems (OR 3.2, 95% CI 1.2-8.3; *P*=.02).

Background factors significantly associated with aberrant motor behaviors were greater impairment in ADLs (OR 1.3, 95% CI 1.1-1.6; *P*=.01), higher K-MMSE score (OR 1.2, 95% CI 1.0-1.3; *P*=.048), older age (OR 0.9, 95% CI 0.8-1.0; *P*=.02), high extroversion traits (OR 0.7, 95% CI 0.5-0.9; *P*=.01), and taking sedatives (OR 0.2, 95% CI 0.0-0.7; *P*=.02).

#### Sleep and Nighttime Behaviors

Among proximal factors, greater numbers of total nighttime sleep hours measured by actigraphy were significantly associated with decreased likelihood of sleep and nighttime behaviors occurring the next day (OR 0.9, 95% CI 0.8-1.0; *P*=.005). In contrast, patients were more likely to exhibit sleep and nighttime behaviors given sleep disturbance observed and reported by caregivers (OR 19.4, 95% CI 11.6-32.7; *P*<.001), urination or bowel movement problems (OR 7.7, 95% CI 4.0-14.7; *P*<.001), or hunger or thirst (OR 4.6, 95% CI 2.2-9.6; *P*<.001). Increased odds of sleep and nighttime behaviors were also associated with environmental change (OR 5.9, 95% CI 2.4-14.6; *P*<.001), inadequate temperature (OR 4.0, 95% CI 1.6-10.2; *P*=.003), and noise (OR 2.7, 95% CI 1.0-6.8; *P*=.04). None of the background factors were significantly associated with the likelihood of sleep and nighttime behaviors.

#### Appetite or Eating Disorders

Of proximal factors, increased likelihood of appetite or eating disorders was associated with exposure to environmental change (OR 11.1, 95% CI 4.2-29.3; *P*<.001), inadequate lighting (OR 3.4, 95% CI 1.1-10.8; *P*=.03), and inadequate temperature (OR 2.9, 95% CI 1.1-8.0; *P*=.04). Patients were also more likely to exhibit appetite or eating disorders on the same day if they experienced hunger or thirst (OR 8.5, 95% CI 4.0-18.1; *P*<.001) or urination or bowel movement problems (OR 6.0, 95% CI 2.9-12.6; *P*<.001). Interpersonal triggers also predicted an increased likelihood of appetite or eating disorders (OR 2.2, 95% CI 1.0-4.6; *P*=.04). The openness trait was the only background factor that significantly predicted appetite or eating disorders (OR 1.3, 95% CI 1.1-1.6; *P*=.003).

## Discussion

### Principal Findings

Although BPSD are recognized to arise from background and proximal factors, it is unclear exactly how the different BPSD subsyndromes are influenced by specific proximal factors, such as sleep and physical activity levels and physical and psychosocial unmet needs states. This study explored factors associated with BPSD subsyndromes among community-dwelling older adults with dementia, including sets of background and proximal factors (ie, actigraphy-measured sleep and physical activity levels and diary-based caregiver-perceived symptom triggers) guided by the NDB model. This research expanded upon the limited previous research by using actigraphy and diary-based assessments, thereby enabling the collection of objective and/or continuous time-varying proximal data.

### The Effects of Proximal Factors on the Occurrence of BPSD Subsyndromes

Our results demonstrated that BPSD subsyndromes were predictable based on proximal factors, including actigraphy-measured sleep and activity levels and diary-based caregiver-reported symptom triggers. The models to predict BPSD subsyndromes demonstrated better predictive ability when both background and proximal factors served as predictors in the models compared to those with only background factors. While BPSD have been largely considered unpredictable [17] and few previous studies have considered the impact of preventative approaches to BPSD [11], our results support a paradigm shift to an individualized approach to BPSD subsyndromes through prediction and early prevention. In particular, caregiver-reported physical, psychosocial, and environmental triggers largely influenced most subsyndromes, except for euphoria or elation. This suggests that, despite the unmodifiable nature of background factors, most BPSD can be prevented through early identification of at-risk individuals and timely assessment of targeted physical and psychosocial unmet needs.

However, it remains unclear as to which BPSD subsyndromes are under circadian control (ie, influenced by sleep and activity levels). In this study, greater nighttime sleep hours were associated with a lower likelihood of sleep and nighttime behaviors, manifested as nighttime awakening or excessive daytime napping, occurring the next day. This finding is consistent with previous studies, in that sleep problems are both a risk factor and a symptom outcome [66,67]. In previous studies, the association between sleep disturbance and BPSD has been inconsistent and variable among different BPSD. For example, a previous study of a Korean sample found that more severe sleep impairment, measured by the Korean version of the Pittsburgh Sleep Quality Index (PSQI) [68], was significantly associated with apathy or indifference, but not with other types of symptoms [69]. In another study conducted in China, the total scores of the PSQI and its subscales were significantly correlated with depression, apathy, and sleep and nighttime behaviors [35]. Moreover, one study of a hospitalized sample noted that the average number of sleep minutes significantly predicted agitation and irritability [37]; another study of nursing home residents with severe dementia found that a greater number of nighttime sleep hours was a predictor of daytime aggressive behaviors [70]. One possible explanation for the nonsignificant relationship between sleep parameters and aggression in our study could lie in the differences in sample characteristics. Given that the mean K-MMSE score in our sample indicated moderate cognitive impairment, nighttime sleep perhaps affects aggression when cognitive impairment is severe. Regarding the activity level, euphoria or elation was associated with energy expenditure measured by actigraphy. A previous study showed that an increased physical activity level was associated with an increased likelihood of agitation and aggression in hospitalized patients with severe dementia [45]. This may be because increased physical activities could reflect heightened agitation and aggressive symptoms per se rather than physical exercise. To summarize, it still remains unclear as to which BPSD subsyndromes are under circadian control. The inconsistent results among prior studies call for further research to build on extant knowledge.

### Implications for Different Approaches to Heterogeneous BPSD Subsyndromes

While the NDB model explains the effect of the interplay between background and proximal factors on the broad construct of BPSD [71], the findings from this study extend the NDB model by indicating which specific background and proximal factors exert their effects on which specific BPSD subsyndromes. For example, while a greater number of nighttime sleep hours was associated with attenuated likelihood of sleep and nighttime behaviors occurring the next day, nighttime sleep hours did not influence other BPSD subsyndromes. While physical unmet needs states (eg, pain or discomfort, sleep disturbance, and hunger or thirst) and physical environmental conditions (eg, noise and lighting) were significant factors associated with affective symptoms, they were not found to influence symptoms of euphoria or elation. Instead, actigraphy-measured energy expenditure was a proximal factor that predicted euphoria or elation, whereas it did not influence any other symptoms. Thus, the results underscore that etiological-based differentiated interventions are needed to focus on the factors underlying the target BPSD subsyndromes [17].

Accordingly, our study provides empirical evidence regarding the heterogeneity of BPSD [13-15] by revealing the distinct underlying predictors for the seven BPSD subsyndromes. Results showed that there were multiple predictors of all BPSD; however, the mechanisms underlying how a set of neurobiological, psychosocial, and environmental factors interplay and subsequently result in specific symptoms remain elusive [72]. Since the etiopathogenesis of BPSD is complex, multifactorial, and variable depending on the BPSD subsyndromes or individual symptoms [12,72], it is challenging for health care providers and caregivers to decide which interventions should be used to manage certain types of symptoms. Several interventional algorithms have been proposed to simplify complex information on the relationships among multifactorial factors and make it usable, such as the Describe, Investigate, Create, and Evaluate (DICE) approach [11,73] and the BPSD–Describe and Measure, Analyze, Treat, and Evaluate (DATE) algorithm [72]. Although the algorithms have been helpful for caregivers to simplify the complex nature of heterogeneous BPSD trajectories, further research is needed to develop predictive models that will guide health care providers and caregivers to move toward personalized BPSD care. Our study represents a starting point for developing more data-driven predictive algorithms tailored to BPSD subsyndromes to advance more precise BPSD care.

### Advancing BPSD Research and Practice by Leveraging Digital Health Technologies

The results also facilitate the use of technological advances to achieve personalized BPSD care. Assessment of BPSD has largely relied on information reported by informal or formal caregivers, which has resulted in information bias and caregiver burden [74]. While the traditional nomothetic approach to assessment of BPSD has limitations in terms of capturing the unique trajectories of BPSD manifestations and their contexts, human behavior and psychology research has considered idiographic approaches to capture prospective and time-dependent variation within individuals [75]. Our study demonstrates the potential of using real-world data to measure fluctuating contextual information by leveraging digital health technologies to advance BPSD research and practice using an idiographic and personalized approach. Although concerns may exist regarding applying actigraphy to patients with dementia, our results demonstrated the feasibility of assessing sleep and activity levels using actigraphy in individuals with dementia, as evidenced by relatively low attrition rates and low amounts of missing data.

Along with the objectively measured actigraphy data, the results of the GLMMs revealed the large magnitude of the associations between diary-based caregiver-perceived symptom triggers and BPSD subsyndromes, which indicates the importance of collateral information from caregivers for predicting BPSD subsyndromes. Information and communications technologies (ICTs) can be a solution to complement paper-based symptom diaries by allowing the collection of high-frequency contextual information on BPSD that is episodic and evolves over time [76]. Future studies could adopt mobile app–based symptom diaries, which would facilitate the tracking and monitoring of BPSD by using push notifications to ensure measurements are not missed and to assist caregivers in easily checking symptoms anywhere and anytime with their mobile phones. Ecological momentary assessment (EMA) [77] is a promising tool that could be incorporated into mobile app–based symptom diaries. EMA data collected using electronic diaries could capture the momentary aspects of BPSD occurrence, rather than relying on recall over time and how BPSD manifestations vary over time and situations through repeated assessments [77]. Moreover, integrating prediction models into digital devices, such as smartphones, or connecting them to electronic health records could further improve the clinical utility of prediction models.

### Limitations

Several limitations should be noted. First, we used a proxy measure of BPSD based on caregivers’ reporting, rather than direct observation of symptoms, which may have introduced observer and recall bias. Second, because BPSD and caregiver-reported contributing factors were checked on a daily rather than episodic basis, caregivers could check multiple types of symptoms and related triggers if different types of symptoms were observed within a day. This may have made it difficult to identify the caregiver-reported factors that contributed to specific types of BPSD that occurred during the day. Future mobile app–based symptom diaries that collect episodic data would make it possible to disentangle aggregated proximal factors and symptoms that occur within a day. Finally, although it was not within the scope of this study, the bidirectionality of the relationships between proximal factors and BPSD is unclear in the current literature. A more thorough understanding of the reciprocal effects of sleep and physical activity levels with BPSD is needed to understand the mechanisms that underly the associations.

### Conclusions

In conclusion, this prospective study demonstrated that BPSD are clinically heterogeneous, and their occurrence can be predicted by different contributing factors. Our results for various BPSD suggest a critical window for timely intervention and care planning. These findings represent the starting point for the establishment of a prediction model tailored to specific symptoms. However, further studies with larger samples are needed to confirm these findings. Nonetheless, these findings will assist in devising symptom-targeted and individualized interventions to prevent and manage different types of BPSD and to ultimately facilitate personalized dementia care.

## Appendix

Multimedia Appendix 1 Background characteristics of older adults with dementia, summary statistics of behavioral and psychological symptoms of dementia (BPSD) and proximal factors, and summary statistics for the prevalence of BPSD subsyndromes.

Multimedia Appendix 2 Summary statistics for the prevalence of behavioral and psychological symptoms of dementia (BPSD) subsyndromes.

Multimedia Appendix 3 Results of generalized linear mixed models (GLMMs) for psychotic, affective, and hyperactivity symptoms and euphoria or elation (Model 1) and results of GLMMs for aberrant motor behaviors, sleep and nighttime behaviors, and appetite or eating disorders (Model 1).

Multimedia Appendix 4 Results of generalized linear mixed models (GLMMs) for psychotic, affective, and hyperactivity symptoms and euphoria or elation (Model 2, full model) and results of GLMMs for aberrant motor behaviors, sleep and nighttime behaviors, and appetite or eating disorders (Model 2, full model).

## Abbreviations

ADL: activity of daily living
AIC: Akaike information criterion
BFI-K: Korean version of the Big Five Inventory
BIC: Bayes information criterion
BPSD: behavioral and psychological symptoms of dementia
DATE: Describe and Measure, Analyze, Treat, and Evaluate
DICE: Describe, Investigate, Create, and Evaluate
EMA: ecological momentary assessment
GLMM: generalized linear mixed model
ICT: information and communications technology
K-ADL: Korean version of the Activities of Daily Living scale
K-MMSE: Korean version of the Mini–Mental State Examination
MMSE: Mini–Mental State Examination
NDB: need-driven dementia-compromised behavior
NRF: National Research Foundation of Korea
OR: odds ratio
PLST: progressively lowered stress threshold
PSQI: Pittsburgh Sleep Quality Index
STROBE: Strengthening the Reporting of Observational Studies in Epidemiology
TST: total sleep time
WASO: wake time after sleep onset
