# Supplementary material for: Factors Associated With Behavioral and Psychological Symptoms of Dementia: Prospective Observational Study Using Actigraphy
Source: J Med Internet Res. 2021 Oct 29;23(10):e29001. doi: 10.2196/29001 (PMC8590188; doi:10.2196/29001)
Supplement: Multimedia Appendix 1 [file jmir_v23i10e29001_app1.docx]

Multimedia Appendix 1

**Table S1.** Background characteristics of older adults with dementia

| Variables | | First wave (N = 145) | Second wave (N = 59) | *p*-value |
| --- | --- | --- | --- | --- |
|  | |  |  |  |
| **Age, year** |  | 81.23±6.01 | 80.10±6.09 | 0.228 |
| **Gender** |  |  |  | 0.876 |
|  | Male | 59 (40.7) | 25 (42.4) |  |
|  | Female | 86 (59.3) | 34 (57.6) |  |
| **Marital status** |  |  |  | 0.271 |
|  | Married | 86 (59.3) | 40 (67.8) |  |
|  | Bereaved or divorced | 59 (40.7) | 19 (32.2) |  |
| **Education** |  |  |  | 0.917 |
|  | Elementary school or below | 73 (50.3) | 28 (47.5) |  |
|  | Middle school | 14 (9.7) | 7 (11.9) |  |
|  | High school | 34 (23.4) | 13 (22.0) |  |
|  | College or above | 24 (16.6) | 11 (18.6) |  |
| **Total ADL score** |  | 10.57±3.63 | 11.03 ± 3.63 | 0.411 |
| **Total MMSE score** |  | 17.28±5.51 | 16.56 ± 5.86 | 0.410 |
| **BFI** |  |  |  |  |
|  | Openness | 8.60±2.96 | 7.97 ± 3.02 | 0.169 |
|  | Conscientiousness | 11.66±2.73 | 11.49 ± 3.18 | 0.712 |
|  | Neuroticism | 7.74±2.87 | 7.42 ± 2.55 | 0.466 |
|  | Extroversion | 8.49±1.87 | 8.31 ± 2.17 | 0.543 |
|  | Agreeableness | 10.92±2.96 | 10.83 ± 2.87 | 0.837 |
| **Sedative, yes** |  | 51 (35.2) | 19 (32.2) | 0.746 |
| **Dementia type** |  |  |  |  |
|  | Alzheimer disease | 71 (49.0) | 21 (35.6) | 0.090 |
|  | Lewy body dementia | 60 (41.4) | 25 (42.4) | 1.000 |
|  | Vascular dementia | 23 (15.9) | 11 (18.6) | 0.680 |
|  | Other dementia | 31 (21.4) | 18 (30.5) | 0.206 |

***Note***. Data are expressed as mean ± standard deviation or number (percentage).

Abbreviations: N, the number of participants; ADL, activities of daily living; MMSE, Mini-Mental State Examination; BFI, the Big Five Inventory.

| **Table S2.** Summary statistics of BPSD and proximal factors | | | | | |
| --- | --- | --- | --- | --- | --- |
| Variables | | Total (N = 2,354) | First wave (N = 1,707) | Second wave (N = 647) | *p*-value |
| **Days of recording BPSD and actigraphy per person** | | 11.54±3.50 | 11.77±3.35 | 10.97±3.83 | 0.161 |
| **BPSD** |  |  |  |  |  |
|  | Psychotic symptoms | 234 (9.9) | 159 (9.3) | 75 (11.6) | 0.105 |
|  | Affective symptoms | 548 (23.3) | 436 (25.5) | 112 (17.3) | <.001 |
|  | Hyperactivity | 350 (14.9) | 275 (16.1) | 75 (11.6) | 0.006 |
|  | Euphoria/elation | 108 (4.6) | 91 (5.3) | 17 (2.6) | 0.004 |
|  | Aberrant motor behavior | 103 (4.4) | 55 (3.2) | 48 (7.4) | <.001 |
|  | Sleep and nighttime behavior | 275 (11.7) | 201 (11.8) | 74 (11.4) | 0.886 |
|  | Appetite/eating disorders | 193 (8.2) | 139 (8.1) | 54 (8.3) | 0.867 |
| **Proximal factors** |  |  |  |  |  |
|  | TST (hours at night) | 6.44±2.52 | 6.51±2.51 | 6.25±2.52 | 0.025 |
|  | WASO (hours at night) | 0.43±0.36 | 0.41±0.35 | 0.47±0.40 | <.001 |
|  | Energy expenditure  (100 kcal/hour) | 0.21±0.14 | 0.21±0.14 | 0.22±0.14 | 0.666 |
|  | Hunger or thirst | 149 (6.3) | 85 (5.0) | 64 (9.9) | <.001 |
|  | Urination or bowel movement | 220 (9.3) | 162 (9.5) | 58 (9.0) | 0.751 |
|  | Pain or discomfort | 190 (8.1) | 139 (8.1) | 51 (7.9) | 0.866 |
|  | Sleep disturbance | 276 (11.7) | 218 (12.8) | 58 (9.0) | 0.010 |
|  | Noise | 76 (3.2) | 55 (3.2) | 21 (3.2) | 1.000 |
|  | Light | 69 (2.9) | 50 (2.9) | 19 (2.9) | 1.000 |
|  | Temperature | 103 (4.4) | 63 (3.7) | 40 (6.2) | 0.013 |
|  | Interpersonal trigger | 171 (7.3) | 124 (7.3) | 47 (7.3) | 1.000 |
|  | Environmental change | 87 (3.7) | 71 (4.2) | 16 (2.5) | 0.066 |
|  | Other | 238 (10.1) | 208 (12.2) | 30 (4.6) | <.001 |

***Note***. Data are expressed as mean ± standard deviation or number (percentage).

Abbreviations: N, the total number of days in which outcome and proximal factors were measured; BPSD, behavioral and psychological symptoms of dementia; TST, total sleep time; WASO, wake time after sleep onset.

| **Table S3.** Summary statistics for the prevalence of BPSD subsyndromes. | | | | |
| --- | --- | --- | --- | --- |
|  | ^a^Total (N = 145) | First wave (N = 145) | Second wave (N = 59) | *p*-value |
| ***BPSD*** |  |  |  |  |
| Psychotic symptoms | 41(28.3) | 41 (28.3) | 10 (16.9) | 0.130 |
| Affective symptoms | 82(56.6) | 73 (50.3) | 27 (45.8) | 0.661 |
| Hyperactivity | 70(48.3) | 63 (43.4) | 15 (25.4) | 0. 025 |
| Euphoria or elation | 37(25.5) | 32 (22.1) | 6 (10.2) | 0.075 |
| Aberrant motor behavior | 25(17.2) | 19 (13.1) | 9 (15.3) | 0.857 |
| Sleep and night-time behavior | 63(43.4) | 54 (37.2) | 18 (30.5) | 0.453 |
| Appetite or eating disorders | 40(27.6) | 35 (24.1) | 12 (20.3) | 0.689 |

***Note***. Data are expressed as number (percentage).

^a^The prevalence of BPSD subsyndromes for the total of 145 participants were calculated using the wave 1 and wave 2 data collapsed.

**Abbreviations:** BPSD, behavioral and psychological symptoms of dementia.
