# Supplementary material for: Factors Associated With Behavioral and Psychological Symptoms of Dementia: Prospective Observational Study Using Actigraphy
Source: J Med Internet Res. 2021 Oct 29;23(10):e29001. doi: 10.2196/29001 (PMC8590188; doi:10.2196/29001)
Supplement: Multimedia Appendix 2 [file jmir_v23i10e29001_app2.docx]

Multimedia Appendix 2

| **Table S1.** Summary statistics for the prevalence of BPSD subsyndromes. | | | | |
| --- | --- | --- | --- | --- |
|  | ^a^Total (N = 145) | First wave (N = 145) | Second wave (N = 59) | *p*-value |
| ***BPSD*** |  |  |  |  |
| Psychotic symptoms | 41(28.3) | 41 (28.3) | 10 (16.9) | 0.130 |
| Affective symptoms | 82(56.6) | 73 (50.3) | 27 (45.8) | 0.661 |
| Hyperactivity | 70(48.3) | 63 (43.4) | 15 (25.4) | 0. 025 |
| Euphoria/elation | 37(25.5) | 32 (22.1) | 6 (10.2) | 0.075 |
| Aberrant motor behavior | 25(17.2) | 19 (13.1) | 9 (15.3) | 0.857 |
| Sleep and night-time behavior | 63(43.4) | 54 (37.2) | 18 (30.5) | 0.453 |
| Appetite/eating disorders | 40(27.6) | 35 (24.1) | 12 (20.3) | 0.689 |

***Note***. Data are expressed as number (percentage).

^a^The prevalence of BPSD subsyndromes for the total of 145 participants were calculated using the wave 1 and wave 2 data collapsed.

**Abbreviations:** BPSD, behavioral and psychological symptoms of dementia.
