# Supplementary material for: Factors Associated With Behavioral and Psychological Symptoms of Dementia: Prospective Observational Study Using Actigraphy
Source: J Med Internet Res. 2021 Oct 29;23(10):e29001. doi: 10.2196/29001 (PMC8590188; doi:10.2196/29001)
Supplement: Multimedia Appendix 3 [file jmir_v23i10e29001_app3.docx]

Multimedia Appendix 3

**Table S1.** Results of generalized linear mixed models for psychotic, affective, hyperactivity symptoms and euphoria/elation (Model 1)

| Predictor variables | Outcome variables (BPSD subsyndromes) | | | |
| --- | --- | --- | --- | --- |
|  | Psychotic symptoms | Affective symptoms | Hyperactivity  Symptoms | Euphoria/  elation |
| **Background factors** | | | | |
| Wave | 0.4 (0.2-0.8)† | 0.6 (0.4-0.9)* | 0.7 (0.4-1.2) | 0.3 (0.1-0.6)‡ |
| Age | 1.0 (0.9-1.2) | 1.0 (0.9-1.1) | 0.9 (0.8-1.0) | 1.0 (0.9-1.1) |
| Gender, female | 1.4 (0.2-8.3) | 0.1 (0.0-0.6)† | 0.3 (0.1-1.2) | 0.5 (0.2-1.9) |
| Education  (ref. Elementary school or below) |  |  |  |  |
| Middle school | 2.5 (0.5-12.2) | 0.2 (0.0-0.6)† | 0.4 (0.1-1.7) | 0.6 (0.1-2.6) |
| High school | 0.9 (0.2-4.5) | 0.4 (0.1-1.5) | 0.8 (0.2-3.1) | 1.6 (0.5-5.5) |
| College or above | 1.6 (0.2-14.2) | 0.1 (0.0-0.8)* | 0.4 (0.1-2.4) | 0.7 (0.1-3.7) |
| Marital status  (ref. Married) |  |  |  |  |
| Bereavement or divorce | 0.2 (0.0-1.3) | 1.2 (0.3-5.0) | 1.8 (0.5-7.2) | 2.8 (0.9-9.1) |
| Sum of ADL | 1.2 (1.0-1.4) | 1.1 (0.9-1.2) | 1.1 (1.0-1.2) | 0.9 (0.8-1.1) |
| Sum of MMSE | 1.0 (0.9-1.1) | 1.0 (1.0-1.1) | 0.9 (0.9-1.0) | 1.0 (0.9-1.1) |
| BFI |  |  |  |  |
| Openness | 1.1 (0.9-1.3) | 1.1 (0.9-1.2) | 1.1 (0.9-1.2) | 1.0 (0.8-1.1) |
| Conscientiousness | 0.9 (0.7-1.1) | 1.0 (0.9-1.2) | 1.0 (0.8-1.1) | 1.2 (1.0-1.5) |
| Neuroticism | 1.0 (0.8-1.1) | 0.9 (0.8-1.1) | 1.0 (0.8-1.1) | 1.0 (0.8-1.2) |
| Extroversion | 1.0 (0.8-1.2) | 0.8 (0.7-0.9)† | 0.8 (0.7-1.0) | 1.1 (0.8-1.3) |
| Agreeableness | 1.2 (1.0-1.6) | 1.4 (1.2-1.6)‡ | 1.3 (1.1-1.6)† | 0.9 (0.7-1.1) |
| Sedative, yes | 7.3 (1.9-28.5)† | 6.2 (2.6-14.5)‡ | 8.3 (3.3-20.9)‡ | 2.9 (1.1-7.3)* |
| Dementia type |  |  |  |  |
| Alzheimer disease | 2.0 (0.2-15.6) | 1.9 (0.4-9.8) | 2.1 (0.4-9.9) | 3.4 (0.9-13.1) |
| Lewy body dementia | 0.8 (0.2-4.5) | 0.9 (0.2-3.3) | 0.7 (0.2-2.5) | 1.3 (0.4-3.9) |
| Vascular dementia | 0.5 (0.0-6.7) | 3.7 (0.6-22.8) | 3.0 (0.5-17.4) | 1.2 (0.2-5.8) |
| Other dementia | 0.7 (0.1-8.6) | 4.3 (0.6-29.4) | 1.4 (0.2-9.4) | 3.5 (0.7-18.0) |

***Note***. Data are expressed as odds ratio (95% confidence interval); *p-value<0.05; †p-value<0.01; ‡ p-value<0.001. **Abbreviations:** ADL, activities of daily living; MMSE, Mini-Mental State examination; BFI, the big five inventory

**Table S2.** Results of generalized linear mixed models for aberrant motor behaviors, sleep and nighttime behaviors, and appetite/eating disorders (Model 1)

| Predictor variables | | Outcome variables (BPSD subsyndromes) | | |
| --- | --- | --- | --- | --- |
|  |  | Aberrant motor  behaviors | Sleep and nighttime behaviors | Appetite/eating disorders |
| **Background factors** | | | | |
|  | Wave | 5.7 (2.1-15.5)‡ | 0.8 (0.4-1.4) | 0.7 (0.3-1.4) |
|  | Age | 0.9 (0.7-1.0)* | 1.0 (0.9-1.1) | 0.9 (0.8-1.0) |
|  | Gender, female | 0.4 (0.1-2.7) | 0.5 (0.2-1.7) | 0.5 (0.1-3.0) |
| Education  (ref. Elementary school or below) |  |  |  |  |
|  | Middle school | 2.1 (0.3-14.7) | 0.7 (0.2-2.4) | 1.2 (0.2-5.5) |
|  | High school | 1.0 (0.1-8.1) | 0.5 (0.2-1.5) | 0.6 (0.1-2.8) |
|  | College or above | 1.3 (0.1-13.8) | 1.0 (0.2-4.4) | 0.4 (0.1-3.5) |
| Marital status  (ref. Married) |  |  |  |  |
|  | Bereavement or divorce | 3.1 (0.6-17.6) | 1.7 (0.6-5.2) | 1.2 (0.2-5.4) |
| Sum of ADL |  | 1.3 (1.1-1.6)* | 0.9 (0.8-1.1) | 0.9 (0.7-1.1) |
| Sum of MMSE |  | 1.2 (1.0-1.3)* | 1.0 (0.9-1.1) | 0.9 (0.8-1.0) |
| BFI |  |  |  |  |
|  | Openness | 1.1 (0.8-1.3) | 1.1 (0.9-1.2) | 1.3 (1.1-1.6)† |
|  | Conscientiousness | 1.2 (0.9-1.6) | 1.0 (0.8-1.2) | 1.0 (0.8-1.3) |
|  | Neuroticism | 0.9 (0.7-1.1) | 1.1 (0.9-1.3) | 1.1 (0.9-1.3) |
|  | Extroversion | 0.7 (0.5-0.9)* | 1.0 (0.8-1.2) | 0.9 (0.7-1.1) |
|  | Agreeableness | 1.0 (0.7-1.3) | 0.9 (0.7-1.0) | 1.0 (0.8-1.2) |
| Sedative, yes |  | 0.2 (0.0-0.7)* | 1.0 (0.4-2.4) | 1.7 (0.5-5.4) |
| Dementia type |  |  |  |  |
|  | Alzheimer disease | 2.7 (0.3-22.4) | 0.6 (0.2-2.1) | 1.3 (0.2-7.2) |
|  | Lewy body dementia | 0.9 (0.2-4.7) | 0.7 (0.2-2.1) | 1.6 (0.4-6.7) |
|  | Vascular dementia | 1.2 (0.1-12.3) | 2.4 (0.6-9.6) | 2.9 (0.5-18.6) |
|  | Other dementia | 0.2 (0.0-2.9) | 1.1 (0.2-5.5) | 0.9 (0.1-7.4) |

***Note***. Data are expressed as odds ratio (95% confidence interval); *p-value<0.05; †p-value<0.01; ‡ p-value<0.001

**Abbreviations:** ADL, activities of daily living; MMSE, Mini-Mental State examination; BFI, the big five inventory
