# Supplementary material for: Factors Associated With Behavioral and Psychological Symptoms of Dementia: Prospective Observational Study Using Actigraphy
Source: J Med Internet Res. 2021 Oct 29;23(10):e29001. doi: 10.2196/29001 (PMC8590188; doi:10.2196/29001)
Supplement: Multimedia Appendix 4 [file jmir_v23i10e29001_app4.docx]

Multimedia Appendix 4

**Table S1:** Results of generalized linear mixed models for psychotic, affective, hyperactivity symptoms and euphoria/elation (Model 2, full model).

| Predictor variables | | Outcome variables (BPSD subsyndromes) | | | |
| --- | --- | --- | --- | --- | --- |
|  |  | Psychotic symptoms | Affective symptoms | Hyperactivity Symptoms | Euphoria/  elation |
| **Background factors** |  |  |  |  |  |
| Age |  | 1.0 (0.8–1.1) | 1.0 (0.9–1.0) | 0.9 (0.8–1.0)† | 0.9 (0.9–1.0) |
| Gender, female |  | 4.5 (0.7–29.4) | 0.4 (0.2–1.2) | 0.5 (0.2–1.6) | 0.6 (0.1–2.2) |
| Education (ref. Elementary school or below) |  |  |  |  |  |
|  | Middle school | 10.2 (1.5–70.1)* | 0.2 (0.0–0.6)† | 0.4 (0.1–1.7) | 0.5 (0.1–2.7) |
|  | High school | 1.6 (0.3–10.3) | 0.6 (0.2–1.6) | 0.8 (0.2–2.3) | 1.3 (0.4–4.6) |
|  | College or above | 6.2 (0.6–66.0) | 0.4 (0.1–1.3) | 0.4 (0.1–2.0) | 0.5 (0.1–3.3) |
| Marital status (ref. Married) |  |  |  |  |  |
|  | Bereaved or divorced | 0.1 (0.0–1.1) | 1.4 (0.5–3.6) | 2.0 (0.7–6.0) | 4.9 (1.4–17.1)* |
| Sum of ADL |  | 1.3 (1.1–1.7)* | 1.1 (1.0–1.3)* | 1.1 (1.0–1.3)* | 0.9 (0.7–1.0) |
| Sum of MMSE |  | 1.1 (1.0–1.3) | 1.1 (1.0–1.1) | 1.0 (0.9–1.1) | 1.0 (0.9–1.1) |
| BFI |  |  |  |  |  |
|  | Openness | 1.0 (0.8–1.3) | 1.0 (0.9–1.2) | 1.1 (0.9–1.2) | 0.9 (0.8–1.1) |
|  | Conscientiousness | 0.8 (0.6–1.0)* | 1.0 (0.9–1.1) | 1.0 (0.8–1.1) | 1.3 (1.0–1.6)* |
|  | Neuroticism | 1.0 (0.8–1.2) | 1.0 (0.9–1.1) | 1.0 (0.8–1.1) | 1.0 (0.9–1.3) |
|  | Extroversion | 1.1 (0.8–1.5) | 0.9 (0.8–1.1) | 1.0 (0.8–1.2) | 1.1 (0.9–1.5) |
|  | Agreeableness | 1.2 (0.9–1.6) | 1.2 (1.0–1.3)* | 1.2 (1.0–1.4) | 0.9 (0.7–1.1) |
| Sedative, yes |  | 4.6 (1.0–21.1)* | 1.3 (0.6–2.8) | 2.5 (1.1–5.9)* | 1.6 (0.6–4.5) |
| Dementia type |  |  |  |  |  |
|  | Alzheimer disease | 2.9 (0.3–27.1) | 1.7 (0.6–5.0) | 2.2 (0.6–7.7) | 5.7 (1.4–23.6)* |
|  | Lewy body dementia | 0.7 (0.1–4.1) | 1.1 (0.4–2.6) | 0.7 (0.2–1.8) | 1.3 (0.4–4.2) |
|  | Vascular dementia | 0.3 (0.0–4.3) | 2.1 (0.6–7.0) | 1.9 (0.5–7.6) | 1.2 (0.2–6.7) |
|  | Other dementia | 0.4 (0.0–6.8) | 3.2 (0.9–11.9) | 1.0 (0.2–4.6) | 4.1 (0.7–23.6) |
| **Proximal factors** |  |  |  |  |  |
|  | Total sleep time (hours at night) | 1.0 (0.9–1.1) | 1.0 (1.0–1.1) | 1.1 (1.0–1.2) | 0.9 (0.8–1.1) |
|  | WASO  (hours at night) | 1.3 (0.6–2.9) | 0.9 (0.5–1.4) | 0.9 (0.5–1.6) | 0.9 (0.4–2.2) |
|  | Energy expenditure  (100 kcal/hour) | 0.1 (0.0–3.5) | 1.1 (0.1–9.0) | 0.6 (0.1–7.0) | 0.0 (0.0–0.5)* |
|  | Hunger or thirst | 2.6 (1.1–6.4)* | 2.3 (1.2–4.6)* | 4.5 (2.1–9.5)‡ | 1.4 (0.4–4.4) |
|  | Urination or bowel movement | 3.1 (1.4–6.9)† | 3.5 (2.0–6.1)‡ | 4.2 (2.2–8.0)‡ | 1.7 (0.6–4.6) |
|  | Pain or discomfort | 4.4 (1.9–10.3)‡ | 8.6 (4.6–16.4)‡ | 8.8 (4.6–16.9)‡ | 1.6 (0.7–3.9) |
|  | Sleep disturbance | 8.8 (3.6–21.7)‡ | 5.3 (3.3–8.7)‡ | 3.0 (1.7–5.2)‡ | 1.0 (0.4–2.4) |
|  | Noise | 1.9 (0.6–6.0) | 12.1 (4.5–32.4)‡ | 8.5 (3.2–22.6)‡ | 0.3 (0.1–1.2) |
|  | Light | 7.8 (1.5–40.7)* | 5.1 (1.5–17.4)† | 3.0 (0.9–10.6) | 2.4 (0.6–9.8) |
|  | Temperature | 0.9 (0.2–4.7) | 2.9 (1.2–7.1)* | 0.9 (0.4–2.4) | 1.8 (0.4–7.6) |
|  | Interpersonal trigger | 3.8 (1.8–8.3)‡ | 8.4 (4.8–14.6)‡ | 7.6 (4.0–14.4)‡ | 13.0 (6.0–28.4)‡ |
|  | Environmental change | 14.7 (5.3–40.8)‡ | 2.4 (1.2–4.9)* | 4.9 (2.1–11.8)‡ | 19.7 (7.5–51.7)‡ |
|  | Other | 5.6 (2.5–12.4)‡ | 8.1 (4.8–13.7)‡ | 4.9 (2.8–8.7)‡ | 8.1 (3.8–17.0)‡ |

***Note***. Data are expressed as odds ratios (95% confidence interval); **p* < 0.05, †*p* < 0.01, ‡*p* < 0.001; a time variable that indicated wave (wave 1 vs. wave 2) was also included in the models.

**Abbreviations:** ADL, activities of daily living; MMSE, Mini-Mental State Examination; BFI, the Big Five Inventory; TST, total sleep time; WASO, wake time after sleep onset; BPSD, behavioral and psychological symptoms of dementia.

**Table S2:** Results of generalized linear mixed models for aberrant motor behaviors, sleep and nighttime behaviors, and appetite/eating disorders (Model 2, full model).

| Predictor variables | | Outcome variables (BPSD subsyndromes) | | |
| --- | --- | --- | --- | --- |
|  |  | Aberrant motor behaviors | Sleep and nighttime behaviors | Appetite/eating disorders |
| **Background factors** |  |  |  |  |
| Age |  | 0.9 (0.7–1.0)* | 1.0 (0.9–1.1) | 0.9 (0.8–1.0) |
| Gender, female |  | 0.4 (0.1–2.7) | 0.5 (0.2–1.7) | 0.5 (0.1–3.0) |
| Education (ref. Elementary school or below) |  |  |  |  |
|  | Middle school | 2.1 (0.3–14.7) | 0.7 (0.2–2.4) | 1.2 (0.2–5.5) |
|  | High school | 1.0 (0.1–8.1) | 0.5 (0.2–1.5) | 0.6 (0.1–2.8) |
|  | College or above | 1.3 (0.1–13.8) | 1.0 (0.2–4.4) | 0.4 (0.1–3.5) |
| Marital status (ref. Married) |  |  |  |  |
|  | Bereaved or divorced | 3.1 (0.6–17.6) | 1.7 (0.6–5.2) | 1.2 (0.2–5.4) |
| Sum of ADL |  | 1.3 (1.1–1.6)* | 0.9 (0.8–1.1) | 0.9 (0.7–1.1) |
| Sum of MMSE |  | 1.2 (1.0–1.3)* | 1.0 (0.9–1.1) | 0.9 (0.8–1.0) |
| BFI |  |  |  |  |
|  | Openness | 1.1 (0.8–1.3) | 1.1 (0.9–1.2) | 1.3 (1.1–1.6)† |
|  | Conscientiousness | 1.2 (0.9–1.6) | 1.0 (0.8–1.2) | 1.0 (0.8–1.3) |
|  | Neuroticism | 0.9 (0.7–1.1) | 1.1 (0.9–1.3) | 1.1 (0.9–1.3) |
|  | Extroversion | 0.7 (0.5–0.9)* | 1.0 (0.8–1.2) | 0.9 (0.7–1.1) |
|  | Agreeableness | 1.0 (0.7–1.3) | 0.9 (0.7–1.0) | 1.0 (0.8–1.2) |
| Sedative, yes |  | 0.2 (0.0–0.7)* | 1.0 (0.4–2.4) | 1.7 (0.5–5.4) |
| Dementia type |  |  |  |  |
|  | Alzheimer disease | 2.7 (0.3–22.4) | 0.6 (0.2–2.1) | 1.3 (0.2–7.2) |
|  | Lewy body dementia | 0.9 (0.2–4.7) | 0.7 (0.2–2.1) | 1.6 (0.4–6.7) |
|  | Vascular dementia | 1.2 (0.1–12.3) | 2.4 (0.6–9.6) | 2.9 (0.5–18.6) |
|  | Other dementia | 0.2 (0.0–2.9) | 1.1 (0.2–5.5) | 0.9 (0.1–7.4) |
| **Proximal factors** |  |  |  |  |
|  | Total sleep time  (hours at night) | 1.0 (0.8–1.1) | 0.9 (0.8–1.0)† | 1.0 (0.9–1.1) |
|  | WASO  (hours at night) | 0.3 (0.1–1.1) | 1.6 (0.8–3.2) | 0.9 (0.4–1.8) |
|  | Energy expenditure  (100 kcal/hour) | 0.2 (0.0–11.6) | 0.5 (0.0–6.0) | 0.3 (0.0–6.6) |
|  | Hunger or thirst | 3.6 (1.1–11.2)* | 4.6 (2.2–9.6)‡ | 8.5 (4.0–18.1)‡ |
|  | Urination or bowel movement | 3.2 (1.2–8.3)* | 7.7 (4.0–14.7)‡ | 6.0 (2.9–12.6)‡ |
|  | Pain or discomfort | 18.0 (6.9–47.1)‡ | 1.8 (0.9–3.6) | 2.2 (1.0–4.9) |
|  | Sleep disturbance | 6.4 (2.7–14.9)‡ | 19.4 (11.6–32.7)‡ | 1.6 (0.8–3.2) |
|  | Noise | 21.6 (5.0–93.5)‡ | 2.7 (1.0–6.8)* | 2.3 (0.8–6.5) |
|  | Light | 0.1 (0.0–0.8)* | 2.3 (0.8–7.0) | 3.4 (1.1–10.8)* |
|  | Temperature | 5.8 (1.6–21.3)† | 4.0 (1.6–10.2)† | 2.9 (1.1–8.0)* |
|  | Interpersonal trigger | 0.8 (0.2–3.4) | 0.6 (0.3–1.3) | 2.2 (1.0–4.6)* |
|  | Environmental change | 5.6 (1.2–26.0)* | 5.9 (2.4–14.6)‡ | 11.1 (4.2–29.3)‡ |
|  | Other | 1.7 (0.6–4.9) | 2.2 (1.1–4.4)* | 1.3 (0.6–2.9) |

***Note***. Data are expressed as odds ratios (95% confidence interval); **p* < 0.05, †*p* < 0.01, ‡ *p* < 0.001; a time variable that indicated waves (wave 1 vs. wave 2) was also included in the models.

**Abbreviations:** ADL, activities of daily living; MMSE, Mini-Mental State Examination; BFI, the Big Five Inventory; TST, total sleep time; WASO, wake time after sleep onset; BPSD, behavioral and psychological.
